# Personalized Technological Support for Informal Caregivers of Older People with Dementia: A Co-Design Approach Involving Potential End Users and Healthcare Professionals in Three Focus Groups in Italy

**DOI:** 10.3390/healthcare11192640

**Published:** 2023-09-28

**Authors:** Francesca Gris, Barbara D’Amen, Giovanni Lamura, Lucia Paciaroni, Marco Socci, Maria Gabriella Melchiorre

**Affiliations:** 1 Centre for Socio-Economic Research on Ageing, IRCCS INRCA—National Institute of Health and Science on Ageing, 60124 Ancona, Italy; f.gris@inrca.it (F.G.); g.lamura@inrca.it (G.L.); g.melchiorre@inrca.it (M.G.M.); 2 Italian National Institute of Statistics—ISTAT, Via Cesare Balbo 39, 00184 Rome, Italy; 3 Neurology Unit, Centre for Cognitive Disorders and Dementias, IRCCS INRCA—National Institute of Health and Science on Ageing, 60129 Ancona, Italy; l.paciaroni@inrca.it

**Keywords:** older people, dementia, informal caregivers, healthcare professionals, personalized digital devices, technology, co-design, Italy

## Abstract

Informal/family caregivers (ICs) of older people with dementia (PwD) can suffer from depression and burnout. However, digital solutions can potentially provide innovative ways to facilitate care provision. The aim of this study was to analyze the opinions of end users (EUs), i.e., PwD aged 65 years and over and their ICs and healthcare professionals (HPs), on the use of digital technology to support care activities. Qualitative data were collected during the co-design phase of the European project “DemiCare—Personalized support for informal caregivers of people with dementia”. This study focused on the Italian context and included two PwD, three ICs, and seven HPs. Three focus groups were held in April–June 2022. Qualitative data were analyzed using MaxQDA 2020 software. Seven thematic areas were identified: daily activities, care tasks, information needs, support received, relationship with and expectations from technology, functionality of the DemiCare integrated system, and ethical issues. Smart devices seemed to be received positively by ICs and HPs, although older PwD seemed to have difficulty accepting the technology. Overall, despite the low number of participants, it seems important and potentially effective to consider the needs and preferences of PwD during exploratory co-design to allow social interactions with them. This study was not registered.

## 1. Introduction

### 1.1. Background

The population is rapidly aging worldwide; and, in this context, the share of people aged 65 years and over is expected to increase from 9.3% in 2020 to around 16.0% in 2050, surpassing the proportions of both children under 5 years (7.1%) and youth aged 15–24 years (13.7%) [1]. Data from the Italian National Institute of Statistics (ISTAT) indicate that as of 1 January 2023, in Italy, where the present study was carried out, the population over 65 years represented 24% of the total population [2], which is the highest proportion among European countries (European Union average: 21%) [3]. Aging is particularly challenging when people become frail, vulnerable, or disabled, and they have difficulties in performing daily activities [4]. This is especially true for older people with dementia (PwD), i.e., with cognitive and behavioral symptoms. The World Health Organization (WHO) estimates that there are over 55 million people with dementia worldwide [5]; it especially affects those aged 65 years and over, reaching a prevalence of 5–10% within this age group in higher-income countries [6]. Further reports [7] estimate that population aging will lead to a great increase in dementia cases globally, from 57.4 million in 2019 to 152.8 million in 2050. In particular, the overall prevalence of dementia among those aged 85 years and over is projected to be 23.5% for men and 30.5% for women in 2050. In the same period, in Italy, the number of PwD is estimated to increase from 1.5 to 2.3 million, i.e., 2.1% of the total population and 9% of those aged 65 or over [8].

It should be pointed out that, overall, dementia affects both older PwD and their informal/family caregivers because, usually, 64% of the former receive assistance at home (mainly from spouses and children), and only 19% and 17% receive assistance at residential care and nursing facilities, respectively [9]. There is, indeed, insufficient provision of public services, and private care is too expensive. In particular, informal care represents about 80% of the total long-term care (LTC) in Europe, with differences among countries, e.g., fewer care responsibilities for relatives and more effective public LTC options in northern areas, a mix of intergenerational solidarity and public cash transfers or in-kind services in continental areas, and the burden of caregiving borne mostly by families in southern countries. This is especially true in Italy [10], where 86% of older people living alone receive care from family members [11], often supported by migrant care workers (MCWs) who work as personal care assistants for seniors [12]. 

However, older PwD and their families can also receive support from digital solutions, which potentially offer innovative ways to provide healthcare services. Using these solutions, care recipients can be safer by being monitored continuously and, thus, can maintain functional autonomy to perform activities of daily living for facilitated independent living. This, in turn, can promote healthy aging, with improved quality of life and overall well-being [13]. Also, digital technology can mitigate the socioeconomic effects of an aging population and can enable aging in place, defined as maintaining independence in one’s place of residence as well as participating in the community during one’s later years [14,15]. Moreover, an informal caregiver (IC) can indirectly benefit from technological support in terms of the psychological aspect and reduced burden [16,17,18]. This is particularly significant because ICs of PwD are considered in the literature as “hidden secondary patients” [19] since they very often experience high levels of anxiety, depression, stress, morbidity, and physical problems and a low quality of life [20,21,22,23]. In Europe, dementia has been, indeed, identified as a “societal challenge”, generating a “necessity for research on technology-related care” [24].

Overall, it appears that there is a need to provide and expand health and social resources to support both PwD receiving care and their respective ICs [7]. This could facilitate the transition of health systems toward the delivery of more integrated care by putting the person at the center and taking advantage of the opportunities offered by digital solutions. In particular, innovative solutions, like telehealth, can reduce the number of hospital readmissions and improve health outcomes [25]. However, several barriers can hinder the deployment of digital solutions in the care sector [16,18]. A scoping review [26] particularly underlined that the acceptance of the technology by PwD depends on several factors, including the attitude of users toward the technology; ethical issues, such as the lack of privacy in remote monitoring; the level of physical and cognitive conditions of those receiving care; and, importantly, the cost. Some barriers to using digital devices are also associated with unrealistic expectations regarding their use or having experienced negative feelings with respect to their use (e.g., frustration, confusion, discomfort, embarrassment, or anxiety), in addition to the low digital literacy of potential end users (EUs) [26].

Based on these factors, there is clearly a need to analyze the perspectives of EUs to develop technologies tailored to their needs. For this reason, several innovative research projects have included a co-design phase with the aim of understanding their preferences and exigencies, and this represents an effective approach to develop “helpful and appreciated technological support” [27]. Some authors [28] have defined “co-design” as the involvement of EUs in the design, delivery, and evaluation of public services. Co-designed solutions have also been highlighted as having a positive impact on health outcomes [29]. In the co-design process, researchers and EUs work together to find the best solutions for developing the final product. Other authors [30] have confirmed that applications (apps) and websites could support ICs; but to develop good-quality online resources, these should be co-designed in collaboration with EUs, who also need to acquire the necessary digital skills to use such tools. When the process involves healthcare professionals (HPs) as well as ICs and PwD (who are both EUs), it creates an atmosphere of common and global knowledge about crucial aspects of living with dementia. On the other hand, most of the interventions that do not consider the perspectives and expectations of EUs may be doomed to fail [31]. However, managing co-design sessions with PwD requires taking into account their cognitive level and their ability to comprehend and communicate [32]. For example, these abilities are maintained in mild cognitive impairment (MCI) and mild dementia (MD). In particular, persons with MCI are “between the cognitive changes of aging and early dementia” [33]; and those with MD, despite objective evidence of low performance in cognitive domains, “retain independence in simpler activities, in contrast to more severe forms of dementia, where basic activities of daily living are compromised” [33]. Moreover, as has been reported by some authors, using a co-design approach remains, overall, important because it makes it possible to “frame common and difficult decisions about dementia care in simple terms” [34].

### 1.2. The DemiCare Project

#### 1.2.1. Study Group and Inclusion/Exclusion Criteria

The results reported in this paper come from the Italian co-design phase of the European research project “DemiCare—Personalized support for informal caregivers of people with dementia’’, as a part of the Horizon 2020 “Active and Assisted-Living Programme” (AAL). The whole study, which started in 2022, is ongoing. The overall project involves four countries: Austria, Italy, Romania, and the Netherlands. It is targeting ICs (aged 18 years and over) and those they care for who live at home and are aged 65 years and over and have MCI or MD (as assessed by Mini-Mental State Examination (MMSE) scores ranging from 20 to 25) [35]. Both the ICs and PwD should be in good overall physical health (e.g., they do not suffer from severe chronic disease, severe disability, oncological disease, or have significant visual and/or hearing impairment), and the carers should have experience using smartphone applications (at least the ability to use an app independently).

#### 1.2.2. Aim and Features of the Project

The aim of DemiCare, by means of a field trial lasting six months and involving 90 caregiving dyads, is to test a personalized digital solution for both monitoring those being cared for and supporting ICs (i.e., spouses, children, and other relatives) using an innovative integrated technological system. In this comprehensive solution, the basic bio-signals and vital parameters of PwD (e.g., sleep patterns and heart activity/heartbeat) are collected by smartwatches worn by the care recipients and smart soles equipped with sensors. The aim of the latter is to measure the pressure distribution and movement of the person’s foot (number of steps, frequency, and speed), thus providing signals for analysis to detect abnormal activities, like wandering, slower walking speeds, longer strides, or variability in strides (all of which allow for gait analysis). All the collected health data are, in turn, elaborated (using standardized interfaces) for a preliminary assessment and transmitted to an innovative DemiCare app installed on the ICs’ smartphones. This mobile application will, thus, support ICs in their interactions with PwD.

#### 1.2.3. Main Outcome: Personalized Support for ICs

To this end, the app requires some preliminary information (gathered by means of validated questionnaires), including the gender, age, education, and health status of both the ICs and PwD; the locality of both and the geographical distance between them; available support networks; care activities provided/received; type of dyad relationship; pre-existing knowledge on dementia; general abilities of the ICs; and the burden of the ICs. Then, an advanced support system will be created by integrating and processing the sociodemographic information of the dyads with the vital data of PwD gained with unobtrusive sensors. This system will provide personalized counselling and training for ICs, so they can make better care decisions, especially with regard to specific/problematic behaviors of older PwD, and approach local/available professionals and peer-support services. In detail, artificial intelligence (AI)-based technologies will extract the relevant socio-health information of the dyad, including possible co-morbidities of the PwD, and will provide personalized guidance to ICs using information/content extracted from specialist literature and presented in a way that is easy to understand. Existing materials will be selected/validated by research teams and then digitized/labeled properly in the app. Available content could include useful topics/articles elaborating on certain topics that can help ICs manage the behavioral changes in PwD (e.g., memory loss, aggression, apathy, depression, inappropriate dressing, and orientation problems/night wandering). Also, further information are provided for the social skills of ICs for interacting with PwD (e.g., verbal and nonverbal), how to create a dementia-friendly environment (e.g., by appropriately rearranging the home), self-care of ICs (e.g., emotional support through support groups and available digital devices), in addition to leisure activities for both members of the dyad (e.g., listening to music/taking a walk/watching television together). The DemiCare app also supports a search function (quick help) for easier access to the contents by entering keywords regarding the topic of interest. ICs will, thus, receive individualized information that meets their specific/personal needs and can be helpful for coping with crucial/relevant symptoms of the PwD and care-related challenges to improve their well-being, e.g., guidance/interventions for increasing the safety of those being cared for, especially in emergency situations, to reduce the ICs’ stress. In addition, unobtrusive/comfortable but effective visual or auditory reminders (ensuring the wearability of the devices) will be developed, as well as strategies so that people will not forget to use them [36].

#### 1.2.4. Overall Novelty of DemiCare

The novelty of the DemiCare study is that it is based on the development of data-driven caregiver support and personalization of information/recommendations, with training/coping strategies to support the ICs of PwD when dealing with specific behavioral symptoms, and prepare them for potential/probable future challenges. Technological solutions have been developed that provide general/overall remote support services for ICs, but these solutions do not seem to consider the individuals’ lack of knowledge or their different needs and skills by not offering individualized guidance [13]. Unlike previous technological solutions, the personalized approach in DemiCare is based on new data gathering methods for dementia-related symptoms via wearable digital devices by combining bio-signals with various care-related and social ecosystem factors. In this respect, the co-design phase, which was not always included in previous studies [31], will enable iterative development. Thus, DemiCare helps to increase caregivers’ knowledge of both dementia and dementia-friendly communication as well as their ability to care for PwD and reduces the psychological, social, and physical impacts related to their role and related responsibilities as caregivers. In turn, this will potentially increase the quality of their relationship with PwD and the safety/quality of care. Moreover, a possible further option and added value of the proposed integrated solution is connectivity with specific features of the public health system (e.g., personal electronic health records), with information made accessible to responsible HPs through certified platforms.

### 1.3. Aim of the Study and Research Questions

The aim of this study, as part of the main DemiCare project, was to better understand the perspectives of EUs (ICs and PwD) and HPs concerning the use of technology for healthcare support in Italy to answer the following research questions: (1) What are the main caregiving activities of the ICs of PwD, and what support is available? (2) What is the current context of the use of digital devices by ICs and PwD, and what is the potential for available technology? (3) What are the opinions/expectations of ICs, PwD, and HPs regarding the potential DemiCare system? (4) How can devices be personalized to improve their acceptance and wearability? The exploration of these aspects and, in particular, the novelty of possibly having individualized guidance for ICs, by means of a preliminary co-creation phase, can highlight the interest of individuals in using digital devices, such as a smartphone app, smartwatch, and smart sole, and their opinions with respect to use and wearability in daily living. This can potentially have an overall positive impact on the design process of the study, in turn, leading to the development of an appropriate and innovative integrated digital system. Furthermore, this makes it possible to overcome the technological research limitations of previous studies involving PwD as well as increase technology acceptance by EUs.

## 2. Materials and Methods

### 2.1. Co-Design Phase

#### 2.1.1. Co-Creation and Focus Groups

The co-design phase of the DemiCare project is the object of this study, as reported in this paper. It was carried out by means of three focus groups in each country of the consortium, with the active involvement of ICs, older PwD, and HPs or other experts in the care sector (e.g., legal/ethics experts). This phase is grounded on the principle that the overall opinions of these actors, in this case, on the general use of technology to support care activities, particularly regarding the potential digital solutions proposed by the DemiCare project, should be acknowledged and addressed, giving special attention to the specific needs of primary EUs [31,37]. Also, creating the right/enabling atmosphere was carefully considered, ensuring that participants would feel able to influence the study outcome by providing them with the correct information [38]. In general, using focus groups in qualitative research [39] is an appropriate way to involve participants in a co-design approach [40], especially with regard to PwD and assisted-living projects, such as interdisciplinary projects on technological innovations that can allow PwD to live at home [41,42], by asking them to provide ideas/opinions in the discussion without having to fill out written questionnaires during individual interviews.

#### 2.1.2. Recruitment of Participants

Participants were recruited in the spring and summer of 2022 in each country of the consortium. Older PwD and their respective ICs were included in two focus groups dedicated to EUs based on a non-probability sampling technique (purposive sampling), with individuals selected for their characteristics (the inclusion criteria mentioned above), which allowed for a good exploration of the themes of the study [43]. They were recruited by contacting public municipal recreational centers and public/private care/assistance institutions. These two focus groups were held in April–May 2022, in Italy (town of Ancona, Marche region), the results of which are reported in this paper. The first was held online; the second, on site to have a more sociable and friendly meeting with them, especially with the latter. Italian EUs were recruited with the help of the Neurology/Alzheimer’s Center/Stroke Unit and the Research Center for Neurological Diseases of Older People at the National Institute of Health and Science on Aging (IRCCS INRCA). In particular, PwD were recruited from among patients who regularly visited the Alzheimer’s disease (AD) daycare center. Eligible subjects (those being cared for and their ICs) were selected with the help of four psychologists, who collected preliminary contacts by providing potential participants with basic information on the study itself. Then, researchers contacted ICs by telephone or e-mail to confirm their participation in the focus groups. HPs in the care sector were then involved in a third online focus group held in Italy in June 2022; they were recruited by the respective research team members, drawing from their own extensive network of relevant expertise.

#### 2.1.3. Data Collection

The two co-design focus groups with ICs and PwD explored the context for potentially using digital solutions (e.g., devices supporting care activities) and the need for information in this regard. They also considered the crucial aspect of unobtrusive but effective reminder mechanisms, ensuring that PwD do not forget to wear the smart devices. In particular, smart devices already in use (e.g., smartphones and tablets) and first impressions of the potential DemiCare app and the overall solution/vision were investigated in terms of the impact on the relationship between the ICs and PwD. In the third focus group, preliminary insights from the previous two were discussed and studied in depth by HPs, focusing on wearability and personalization factors (e.g., the ICs’ background/knowledge of dementia and available local care providers) that could influence what information is needed from different ICs. Moreover, challenges regarding the willingness of PwD to be monitored and to use smart devices (such as a smartwatch or smart sole) were explored. During the three focus groups, slides presenting the integrated DemiCare system and the co-design approach and cards describing potential smart devices were used. The questions asked to participants (topic guide) are presented in Table 1.

### 2.2. Ethical Issues

Participants were provided with detailed information in the form of descriptive material, or easy-to-read guidelines [44], on the aims, contents, methods, procedures, and timing of the overall study and, in particular, the co-design phase, including information on data use. All the participants signed a written informed consent form prior to data collection, including permission to audio-record the narratives. Reassurance regarding anonymity and the privacy/confidentiality of personal information was also provided. To protect the personal data of the participants, their sensitive data (name, surname, address, and telephone number) were reported exclusively on a contact list, where each person was identified with an alphanumerical code (identification number). This list was maintained only in electronic format, with appropriate protection measures (secret access key/password), accessible only by the research team. The provision of written informed consent was considered as sufficient for the co-design step (April–June 2022), as proposed in a previous study [45] because at this stage, “the participant is not someone on whom research is ‘done’, but who is actively engaged in designing and implementing the research process” [46]. The overall study was conducted in accordance with the Declaration of Helsinki (2013) and the ethical issues indicated by EU General Data Protection Regulation (GDPR) no. 679 of 27 April 2016 [47]. Moreover, for the overall study in Italy and the related trial, ethical approval (CE INRCA 22020, DET. no. 571 dated 25 November 2022) was further obtained from the Ethics Committee of IRCCS INRCA. It is also necessary to specify that, with regard to the ICs and PwD involved in the second focus group, the signatures of both on the same informed consent form were required, which also served to reassure the PwD that the ICs were “close” to them during this experience. This approach was also approved for the overall study by the Ethics Committee of IRCCS INRCA.

### 2.3. Data Analysis

During the three focus groups held in Italy, all the narratives were audio-recorded, anonymized, and transcribed verbatim. The qualitative approach for the analysis followed the narrative research according to the constructivist research paradigm, whereby “knowledge of the observed is constructed rather than discovered” [48]. In addition, an open coding process was applied [49], and relevant concepts were identified by adopting the constant comparison technique [50].

The transcribed narratives were read by three researchers independently (FG, MGM, and BD), and the contents were codified to highlight concepts raised by the focus groups. At the end of this analytical stage, the researchers compared the outcomes of their independent analysis to identify commonalities and discuss any disagreement. The subthemes that emerged from the analytical process were then grouped into codes referring to the same phenomenon, according to their similarities. These were subsequently grouped into overarching/higher-order themes, which were finally described in a conceptual map [51], to provide a clear description of the findings. Some relevant quotations are included, with codes indicating only the person’s role in the focus group (PwD, IC, or HP) and a serial number, to ensure the deidentification of the excerpts.

The Italian team members were healthcare workers (two psychologists, two gerontologists, and two sociologists) with expertise in overall caregiving of older people. With regard to the trustworthiness of the qualitative analysis results, our study met some fundamental criteria [52]: credibility (the use of a topic guide partly based on questionnaires applied in previous studies on PwD) [53], analytic transferability (a preliminary literature review for background data), dependability and confirmability (a detailed description of the study protocol and the use of replicable methods), and collaborative discussion with colleagues to reach a shared vision on the final coding.

The analysis was conducted with the support of MAXQDA 2020 software (VERBI Software, Berlin, Germany) [54], a commercially available computer-assisted qualitative data analysis software (CAQDAS) package [55], to make the analytical process more flexible and effective [56]. We also followed the Standards for Reporting Qualitative Research (SRQR, Table S1) [57], the guidelines for STrengthening the Reporting of OBservational studies in Epidemiology (STROBE, Table S2) [58], and the Guidance for Reporting the Involvement of Patients and the Public (GRIPP2 checklist short form, Table S3) [59].

## 3. Results

### 3.1. Characteristics of EUs

The first focus group involved two ICs, and the second one involved ICs and their respective PwD. One IC participated in both focus groups, as allowed by the overall study design, to maintain (when possible) the continuity of the collaboration with EUs throughout the whole co-design phase (and the whole project). However, this did not raise the problem of “double counting” the related opinions because the aims of the two focus groups were different, with different questions and answers, as explained above (Table 1). Unfortunately, few PwD and their respective ICs were recruited owing to difficulties we encountered in involving them in the study, as previously reported by others [29,31]; and, thus, the criteria for sampling the saturation in this respect were not applied, which is better explained in the Limitations Section.

The three ICs (two sons and one wife) were aged 51–69 years, married, and living with their respective spouse. They are workers and a housewife. The two PwD are age 73 and 85 years, both retired, one living with their spouse and one living alone, one affected by MD (MMSE 20–25) and one by moderate dementia (MoD; MMSE 10–20) [35]. Even though the eligibility criteria of the study were aimed at including subjects with MD, for the co-design phase it was also considered as useful to consider moderate dementia; thus, a person with MoD was recruited to evaluate possible differences in opinions between the two stages of dementia and provide some confirmation that seniors with MD represent an adequate target for the subsequent trial of the integrated system to test its usability and acceptance by PwD who may require some supervision, support, and assistance. Those with lower MMSE scores may not require daily assistance; and, for those with a higher MMSE score, the DemiCare system would not be suitable. Moreover, overall, the participation was equal in terms of gender.

### 3.2. Characteristics of HPs

The third focus group involved seven HPs (three psychologists, one neuropsychologist, one biomedical engineer, one healthcare social worker, and one medical doctor). It is worth clarifying that HPs in Italy also receive ethical–legal training, especially psychologists and medical doctors, and experts with specific ethical–legal expertise (as allowed by the study with regard to the overall range of possible HPs/experts in the field to be included) did not participate. These seven HPs were, thus, considered sufficiently representative [38], and their main roles and areas of expertise are presented in Table 2.

### 3.3. Main Themes and Subthemes

**Figure 1:**
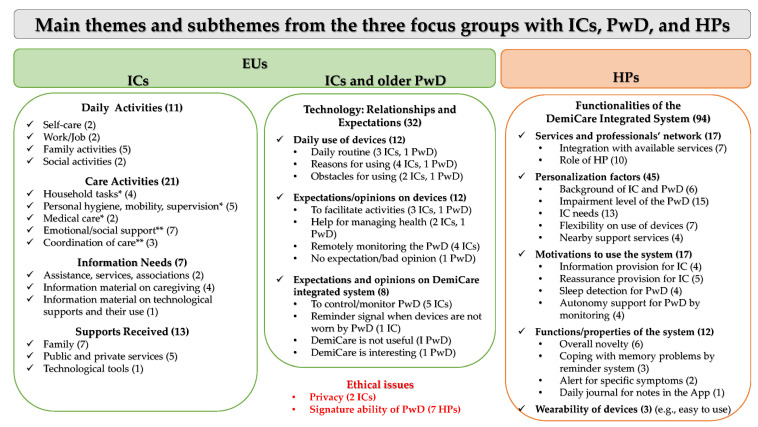
Themes and subthemes that emerged from focus groups. EU, end user; IC, informal caregiver; PwD, people with dementia; HP, healthcare professional; * HB, high burden; ** LB, low burden. Numbers in brackets are numbers of citations/statements of themes/subthemes (including several statements by the same IC/PwD). Statements are listed according to how questions/aspects were proposed and not in decreasing order.

The analysis made it possible to classify the results from the three focus groups into 187 main categories (including several statements by each respondent) related to seven main thematic areas (and further subthemes). Care activities were classified according to the related high burden (HB) or low burden (LB) for ICs, as has been suggested by authors in a previous study [60]. The themes and subthemes are shown in Figure 1.

It is worth clarifying that other topics in addition to ethical issues and opinions on the DemiCare solution were discussed by both the ICs and HPs (e.g., monitoring PwD, especially at night; signals for reminding PwD). However, it was decided to keep the answers separate in Figure 1 because they represented different nuances/expressions, depending on the participant’s role in the focus group. The ethical issues, conversely, were mainly focused on privacy and the ability of PwD to sign informed consent letters to participate in a study; thus, they were proposed as a transversal category.

### 3.4. Focus Group with ICs

From the first focus group, four main themes were identified: general daily activities of ICs, care activities regarding the PwD they cared for, information needs for caregiving, and available support, including technological tools.

The analysis made it possible to identify further subthemes among daily activities: self-care (e.g., eating, showering, and sleep habits), employment activities (own job), and family/social activities (e.g., doing housework and going out for walks). Also, among the care activities, some were highlighted as having a high burden, including household tasks (e.g., doing laundry, preparing meals, and shopping), personal hygiene, mobility and overall supervision in this respect (e.g., bathing, toileting, and help in getting around), and medical care (giving medication, pills, and injections). On the other hand, some care activities were considered to have a low burden, e.g., emotional/social support (providing companionship and facilitating leisure activities) and the overall coordination of caregiving. In particular, participants felt that they did not have enough time for general daily activities and for themselves, owing to demanding care tasks and, subsequently, had difficulty reconciling their own needs and caring for PwD, especially when they made frequent visits in person (when the person being cared for did not live with them) and telephone calls every day.

“I have really very little time for myself!”

(IC-1)

“I am the main support for accompanying her [mother] to the various medical examinations [...] and for managing the bureaucratic procedures.”

(IC-2)

“My mother does not feel comfortable with another person [son] with regard to her personal hygiene.”

(IC-1)

The ICs also reported that they needed information, such as on available care support services and associations with expertise in caring for older PwD. In particular, they wanted materials on care recipient management, as well as possible technological support (and its effective use) that could be of help in this respect.

“It would be important to know the opportunities to get support and assistance, perhaps from public entities.”

(IC-1)

“It would be useful to have information to find an adequate private assistant with expertise in the care of older persons with dementia.”

(IC-2)

“Information on the use of technology in these circumstances could be really helpful.”

(IC-2)

In addition, participants described some practical and emotional support received from family members (e.g., a nephew who was chemist and took care of drug issues and nephews who occasionally accompanied PwD to a medical visit), but this does not seem to be enough because they remain the main/primary caregivers. Moreover, they would benefit from having access to a daycare center for older people, where PwD are engaged in stimulating activities. However, accompanying PwD to such a center might involve further commitment.

“I have no other people around who can help me, just family members, but I am the primary assistant. […] Then, there is the support of the daycare center.”

(IC-1)

“Occasionally my children support me to accompany my mother to the daycare center.”

(IC-2)

In one case, technology seemed to provide some relief, in the form of a video camera installed in the mother’s house that could help, for instance, remind her to turn off the lights when she goes to sleep at night. This IC, however, also noted the possible lack of technological skills that could make the use of digital devices very difficult.

“We have installed a video camera for better monitoring her [mother] and to talk with her. […] However, I do not feel I am skilled enough as regards the overall technology!”

(IC-1)

### 3.5. Focus Group with ICs and Older PwD

From the main theme regarding one’s relationship with and expectations for technology, three main subthemes emerged: the current context of the daily use of smart devices, expectations of technology in general, and expectations of the proposed DemiCare integrated system.

The most common devices used daily by ICs were mainly smartphones, personal computers (PCs)/laptops, and tablets, including the use of social media. In particular, several reasons were reported for using them. They used WhatsApp and Facebook to remain in contact with others; digital calendars, internet banking, and e-mail via smartphone to support some job tasks; and the internet to search for health information. The ICs also used other technological tools, including clock radios, digital weather stations, digital bathroom scales, digital cameras, headphones/headsets, robot vacuum cleaners, and microwave ovens.

“I use the tablet and the smartphone regularly, both at home and outside. I search for various sources of information on the internet.”

(IC-2)

On the contrary, for PwD, some of the symptoms seem to hinder their acceptance and use of technological tools, especially when they are not easy to use; only the person with MD used some devices and considered them useful, such as a smartphone and PC (the latter with the help of the IC/wife), to keep in touch with relatives and to take pictures.

“I use a PC with my wife, and the smartphone alone.”

(PwD-3)

“Currently, the smartphone can do many things; it supports people in many tasks.”

(PwD-3)

“It is not always easy to use technology! […] I use an old basic phone with two buttons, one green to open the calls, one red to close them. I feel I do not need anything else!”

(PwD-2)

In addition, comments on the effective use of technology, personal expectations, and opinions were collected. The ICs expressed a positive opinion of digital devices as instruments that can facilitate several activities (e.g., to obtain useful information on several topics) and can also be helpful for managing one’s own health (e.g., to check blood pressure). They also reported that they would welcome the possibility of remotely monitoring PwD who live alone. In addition, they considered that technological tools would be helpful for guiding PwD in some activities (e.g., remembering to eat breakfast) and that devices could send an alert when older users fall (within or outside their home) or when abnormal vital functions are registered (e.g., an irregular heartbeat).

“Smart devices can facilitate certain daily activities and can also be used for leisure.”

(IC-3)

“Technology could be useful to monitor PwD when they are not close to their family, and the digital device can send an alert in case of falls.”

(IC-2)

However, whereas the older person with MD had a positive opinion of digital devices, the older person with MoD did not appreciate technology at all and thought that simple analog tools were enough.

“Older people need to use simple things!”

(PwD-2)

Finally, the DemiCare integrated system was presented, and the participants (PwD and ICs) shared their feedback with the researchers. The ICs agreed to test the digital solution to track the location of PwD by the Global Positioning System (GPS) and to monitor changes in the physical (falls) and mental (memory impairment) domains. In this respect, they would especially appreciate a system that could register vital parameters during the night, especially for PwD who live alone. They also suggested providing a solution to check whether PwD are wearing the device; that is, the device (e.g., smart sole) would send a reminder signal when it does not register body temperature for a certain period. The signal should be unobtrusive so that PwD will use the device.

“Smart soles with GPS are useful when older people are outside their homes to alert their family for some urgent needs. […] The smartwatch is useful also during the night to continuously monitor blood pressure.”

(IC-3)

“Smart soles could beep in case they have not been worn for a long time, but this beep should not be continuous and annoying.”

(IC-2)

Again, the older person with MoD did not perceive the technology as useful and was not interested in testing the DemiCare system. On the other hand, the older person with MD was very interested, very curious to try it, and asked questions about the study.

“I have never forgotten anything! My old watch is enough! I do not need any smartwatch, just a normal watch is enough. It’s easier.”

(PwD-2)

“I would try the DemiCare system. I would also like to know how many countries work in the study and if there are companies producing smart devices in the consortium.”

(PwD-3)

In addition, both ICs pointed out some ethical issues (transversal category) regarding the possible risk that private data about their vital parameters would “in some way” become available on the internet when they used the integrated solution.

“Smart devices can be of great help, but privacy must be protected.”

(IC-3)

### 3.6. Focus Group with HPs

The focus group with HPs was aimed at exploring the effective and potential functionalities of the DemiCare integrated system (main theme), and five main subthemes emerged: services and professional networks, personalization factors, the motivation to use the system, the functions and properties of the DemiCare app, and the wearability of smart devices.

The HPs indicated that the DemiCare system could first benefit from integration/collaboration with other available support services, thus creating an overall service and professional network (with physicians, psychologists, and healthcare social workers). However, technology cannot totally replace face-to-face contact with HPs (at least sometimes), especially when a diagnosis (and further progress of the disease) must be communicated to the family.

“There is a need for integration with existing services, in order to create a network.”

(HP-1)

“The app works well in association with a professional filter that supports ICs to deal with the situation. I do not think all ICs can fully understand the patient’s behavior and react. […] So, the app is a tool, a support, but it is not adequate by itself.”

(HP-6)

The most appreciated functionality of DemiCare is the personalization of the service, that is, appropriate support for the specific needs of both PwD and ICs. Amid the main personalization factors, the HPs highlighted that the contents of the app for ICs should be adapted to the impairment level/diagnosis of PwD, as well as to the ICs’ needs.

“I think that personalization of digital devices is a very important aspect, since there are different needs.”

(HP-3)

“In the case of MCI, some tools are needed; in the case of MD, other tools could be necessary.”

(HP-2)

Moreover, the background (personal characteristics) of the IC–PwD dyad is considered to be crucial, e.g., their genders, the IC’s occupational status, available support networks, care activities, type of relationship, care burden, and the IC’s knowledge of dementia, including symptoms, such as sleep disorders.

“Information provided by the app, such as actions for managing symptoms and behaviors, could be interpreted differently by ICs, depending on their preparation, according to their character, because the anxious state of a caregiver has an impact on this.”

(HP-4)

“The knowledge that family members have about dementia and its consequences, how it will evolve, is heterogeneous, and the app should start from this consideration.”

(HP-6)

The personalization factors of both the app and devices worn by PwD (e.g., smart soles) also involve the flexibility of use: the more PwD can decide when to wear them and the ICs can decide when to use the app, the more the system would be accepted and perceived as useful.

“For PwD it is not easy to wear prostheses, hearing aids, glasses, and so on. Thus, they should be free to use the devices and to decide when/how long to use them during the day.”

(HP-7)

“Flexibility should be precisely this: caregivers working in the afternoon can watch the app in the morning; caregivers working in the morning can watch it in the evening. People are different: someone could watch the app five times a day; someone else, only once a week.”

(HP-4)

Finally, the information provided by the app could be personalized according to available/nearby support services (e.g., local care providers, hospitals, and AD centers), especially in the case of emergencies. In this respect, the GPS functionality could be helpful to localize these services.

“This app should be a compass that orients people toward existing/nearby support services. A geolocation system would be useful to know what services and possibilities there are in the area where people live, especially in the municipality and in the neighborhood.”

(HP-2)

The motivations that could lead to greater use of the integrated system cover several areas. For example, ICs want support to search for more correct information about diseases and related symptoms or reassurance when they do not know how to deal with behavioral manifestations.

“The information provided by the app is good and validated, and this can convince ICs to use it instead of generally navigating the internet. Information from the internet is not always correct and is not always interpreted in the best way.”

(HP-1)

“The app can be reassuring, especially when ICs need to manage problematic behaviors and when they live far away from PwD. […] So, the information available from the app could support the IC to calm down the person being cared for, somehow.”

(HP-7)

The possible functions allowed by smartphones (e.g., GPS and heart rate and blood pressure monitoring) and smart soles (e.g., gait measurements) serve to maintain the autonomy of older people who can remain at home as long as possible. In addition, when PwD live alone, ICs cannot collect information during the night (e.g., awakening, wandering, and agitation); but, with the support of the DemiCare system, sleep control will be possible.

“The DemiCare system could help to maintain the autonomy of seniors who still live alone for as long as possible, and this is no small thing.”

(HP-5)

“It is important to know what happens during the night because sleep disorders indicate the existence of problems during the day. […] To know this also helps HPs from pharmacological and overall care management points of view.”

(HP-2)

Regarding the properties of the DemiCare solution, the functions that seem useful were explored and, in particular, the more innovative ones compared to what already exists on the market. Apart from a general opinion on the overall novelty of the integrated system (smart soles, smartwatches, and the app), the possibility of being able to cope with the ongoing cognitive impairment of PwD was reported because the system could remind them to perform certain activities (e.g., wash every morning) with visual or audible signals. Then, the system is useful when it sends alerts to the IC regarding symptoms requiring attention (e.g., delirium). The possibility for the app to include a useful daily journal in which ICs could note their own emotional state and monitor their distress level was also appreciated.

“The system could remind PwD to do the activities of daily living, thus solving a practical problem.”

(HP-2)

“It could be useful to put in the app a section reserved for the IC, a virtual diary for writing care problems that might have arisen during the daily routine and to write how the IC solved them.”

(HP-3)

Opinions on the wearability of smart devices by PwD are reported together in Figure 1 because there were only three of them, and they expressed the same meaning. These generally focused on some characteristics of the devices themselves, e.g., ease of use, comfort, dimensions, and battery charging frequency, in addition to personal habits, e.g., habitual wearer of a wristwatch or not.

“The comfort of a device is important; it should be light, easy to use, and charged […]. Moreover, the wearability of a smartwatch could be perceived as greater when PwD already wear a watch daily as something they have always done.”

(HP-5)

Concerning ethical issues (the transversal category), all the HPs noted the crucial importance of establishing whether (and how) PwD can sign informed consent letters when they are involved in an AAL study, like DemiCare. It should also be considered that older people with MCI, as compared with those with a diagnosis of dementia, are still almost autonomous and could refuse to be monitored (even by their ICs).

“Personal and sensitive data are required in this study, so clear informed consent must be given, particularly in the case of a person with a diagnosis of dementia.”

(HP-1)

“Often, an older patient with MCI is not willing to be controlled or controllable.”

(HP-4)

## 4. Discussion

The aim of this study was to involve ICs, the older PwD they care for, and HPs in the co-design phase of the DemiCare research project to explore their potential interest in the use of smart devices to support caregiving activities. Their opinions with respect to the wearability and personalization of devices that could be used in daily living (e.g., smartwatches and smart soles for PwD and a smartphone app for ICs) were explored. Even though the results are from a very small sample, they seem to indicate that smart devices would be positively accepted by ICs and HPs, but limitations in terms of PwD having difficulty accepting technology also emerged, especially the senior with MoD, who was not a member of an official target group of the project but was included in the focus group phase for the reasons explained in the Section 3. According to the literature [29], the use of technological solutions has often been limited owing to a lack of consideration of the needs and opinions of EUs, especially older people, during their development. On the other hand, aspects, such as their perceived usefulness and the safety of the technology, in addition to the individual characteristics of EUs, can make a positive contribution in this respect. Also, some authors [61] have reported that overall, facilitating collaboration among researchers, HPs, and patients in the co-design of relevant information that meets EUs’ needs can potentially have a constructive impact on the development of digital devices. Thus, it is particularly important to involve EUs in the collaborative and participatory design of products and to consider the findings from this approach despite the small number of PwD and ICs involved in this study, as described in detail in the following subsections.

### 4.1. Daily Routine and Care Activities of ICs and Available Support

One crucial aspect that emerged in this respect is that the participating ICs felt that they did not have enough time for general daily activities or for themselves (e.g., self-care, employment, and going out for a walk) owing to demanding care activities, especially those with a high burden (household tasks, personal hygiene, and giving medication), with consequent difficulties in reconciling their own needs with caring for PwD. ICs are, indeed, primary carers; that is, they are family members who take the main responsibility for the extended care of sick older relatives and dedicate many hours per week to caregiving activities [60,62]. This is also because they receive little practical and emotional support from other relatives (e.g., children and sisters/brothers), although they do get some relief during the hours that PwD spend at daycare centers. Several authors have emphasized that family caregivers take care of PwD at home; thus, they experience physical, emotional, and social harms, which also depend on the presence of behavioral problems [63]. Moreover, the caregiving burden often leads to social isolation, with a possible negative impact on work performance [64]. In this context, our respondents reported that they need more information on available care support services, including possible technological tools, although these might be difficult to use for EUs with a low digital competence. The literature widely reports that remote technology-based interventions can be of great benefit for ICs of PwD to manage stressful circumstances, but often ICs are not familiar with them owing to a lack of technological skills, which can make the use of digital devices very difficult [65]. However, ICs first need recommendations, guidance, and more information/educational materials on dementia, especially regarding the related behavioral symptoms and how the disease progresses and worsens, for more appropriate management of care [66,67].

### 4.2. Overall Technology and the DemiCare Integrated System: The Perspectives of ICs and PwD

The ICs who participated in the focus group used a smartphone, PC, or tablet, as well as social media almost daily, mainly to remain in contact with others and to find information on the internet for their own health. They also reported positive opinions on these digital tools, as well as the DemiCare integrated system, especially for recording vital parameters of PwD during the night and to receive alerts when abnormal values are registered. Importantly, devices could remind PwD to perform some activities by means of unobtrusive signals. The results of several studies support these findings, indicating that ICs generally appreciated using technological supports [65], especially when they increase the safety of the PwD that they care for [24]. Digital solutions have the capacity to ease the daily lives of EUs by providing remote health monitoring of PwD, for instance, by detecting if they fall at home [68]. In particular, it was found [29] that older EUs perceive a greater value in technology when it positively impacts their health and well-being in addition to its perceived usefulness [65]. However, digital devices should be placed in an unobtrusive manner; that is, they should require little attention in the EUs’ daily lives, resulting in better acceptance [69,70,71]. Moreover, our ICs reported that they would greatly appreciate having tools with GPS available to track the location of PwD. In this respect, some authors [71] have stressed the importance of such a system because the possibility that PwD may get lost when they are outside alone is a great fear of their respective carers. Those authors also found that ICs recommended the use of GPS installed on a tracking device for PwD, and almost half of those who used such devices “experienced more freedom and were less worried when they were outside unaccompanied” [72]. Other authors have reported that tracking technology increases the perception of independence for both ICs and the relatives that they care for [73,74].

However, these were PwD at an early stage of the disease, and their caregivers could integrate the use of the system into their daily routines [72]. A similar situation could also be confirmed by the PwD who participated in our focus group because only the one with MD reported appreciating being able to use digital tools (smartphone and PC) to take pictures and keep in touch with relatives, and he was also interested in testing the DemiCare solution. Conversely, the senior with MoD did not perceive the overall technology as being useful, considered it difficult to use smart devices, and was not interested in the DemiCare integrated system. The stage of dementia and related symptoms, thus, may be a barrier to using and accepting technological tools, especially when they are not easy to use. In this respect, a previous study [75] reported that the clinical application of telecommunication technology was feasible with patients with AD at its very early stage. Also, it was found [76] that seniors in an early stage of dementia could learn and benefit from user-friendly technology. Other authors have reported the adoption of technology in dementia care as feasible and acceptable for people living with MD and their carers [77]. Technology-based interventions need to be perceived as useful as well as simple and practical to use, according to the personal capabilities of EUs, to be accepted by both carers and those being cared for [24,64,74,78]. It is also worth noting that PwD are antagonistic toward digital tools, especially when they are not used for leisure and social interaction [65].

### 4.3. Functionalities and Properties of the DemiCare Integrated System: The Perspective of HPs

In the focus group with the HPs, some aspects that had already emerged were reinforced, and further issues were proposed. The HPs stressed that the DemiCare system could benefit from being integrated in an overall network of available services and socio-healthcare professionals because they could not be totally replaced by technology. Accordingly, previous studies have pointed out that technology cannot substitute for “care and cure” in person [24], that effective collaboration among professionals is necessary to support the decision-making process [72,79], and that ICs should be integrated into existing healthcare and support services [64].

Our HPs also emphasized the need for personalization to provide solutions that meet the specific characteristics and needs of EUs. Several authors have noted that the findings from research will be “the same” if the individuality of EUs is not considered [24]. Effective technology-based interventions, indeed, require personalized features [80] because PwD and their ICs have different problems and preferences; thus, different solutions should be provided [72], which should also take into account individual attitudes toward the technology and experiences with it [24,81]. Regarding personalization, our HPs also suggested that the contents of the app be adapted to the level and type of dementia and, more generally, to the personal background of the IC–PwD dyad (e.g., sociodemographic characteristics) in accordance with previous studies [24,81].

More recently, it has been highlighted [82] that a “one-for-all” app was not the best solution for supporting ICs of PwD because ICs have different backgrounds, knowledge of dementia and technology, and needs for information and support. Our HPs added flexibility to the time of use of digital devices and more information on nearby support services (e.g., local care providers) as further crucial aspects of personalization. Other authors have pointed out that EUs should be free to use digital tools whenever they want and whenever they decide to connect with them [72]. Another study [83] stressed the importance of providing personalized support for older people by allowing access to update information with a digital ecosystem that links healthcare domains and technology.

As for the motivations for using the DemiCare system, the HPs indicated the ability to search for certified and reassuring information about the disease and related symptoms in the app and to help those being cared for to maintain their own autonomy for as long as possible, especially at night, by means of appropriate monitoring systems. Accordingly, some authors [65] have framed technology as a solution that can enable PwD to live almost independently, at least to a certain stage of the disease, which would also provide ICs with additional personal time away from caregiving. Other authors [64] have noted that technology can improve both the efficiency and effectiveness of formal caregivers and ICs by enhancing the autonomy of those they care for and avoiding their early decline. Regarding the aspect of reassurance for ICs, it has been reported [24] that technology for surveilling PwD increases the reliance and peace of mind of ICs, thus reducing their burden. In this respect, other authors have noted an increased sense of reassurance from adopting technology for both ICs and those they care for [73,74].

As for the functions and properties of the DemiCare solution, apart from the innovation of integrating more digital devices (smart soles, smartwatches, and the app), the HPs who participated in the focus group indicated that it would be very important to enable ICs to deal with the progressive cognitive impairment of PwD with the use of signals to remind them to perform basic daily activities and alerts on symptoms to look out for. Some authors have similarly reported that PwD may use digital devices more routinely when they are relevant for daily activities, such as walking and washing [24,84]. Other authors [73] have reported positive outcomes from empirical studies on reminder devices and memory aids from EUs’ experiences, with a good impact on caregivers’ psychological burden, depression, and anxiety, in particular, for daily activities regarding healthcare [85], e.g., for reminding care recipients to take their medications [86].

Regarding the wearability of smart devices for PwD, our HPs stressed aspects, such as ease of use, comfort, dimensions, and battery charging frequency, in addition to the person’s individual habits, e.g., whether they wear a wristwatch habitually. Previous studies have also focused on the acceptance of technology as measured by perceived usefulness, perceived ease of use, and behavioral intention [87]. With particular regard to PwD, studies [24,84] have pointed out the importance of devices having a small size and light weight in addition to a long battery life (e.g., using a lithium-ion battery) to avoid constant charging. Moreover, it has been found [24] that PwD will tend to dispose of a digital device if they have never used it before, and ICs prefer wristband- or watch-sized devices [78]. Some authors [88] have also described the concept of wearable and unobtrusive technology that can detect vital health parameters (e.g., blood pressure, heart rate, and respiratory rate) and provide timely information on the health condition of chronic patients, thus representing appropriate techniques for long-term monitoring. More recently, findings from focus groups conducted with ICs, PwD, stakeholders, and home care professionals to identify wearable and unobtrusive monitoring technology in home care for dementia [89] have revealed that stakeholders, in particular, indicated organizational collaboration, among other aspects, as an important prerequisite for effective implementation in this regard. Similarly, our HPs generally stressed collaboration among professionals as a key benefit for the overall effective performance of the DemiCare integrated system.

### 4.4. Ethical Issues and Privacy When Older PwD Are Involved

Both the ICs and HPs pointed out some ethical issues regarding the use of digital devices. In particular, the former stressed the need to protect the privacy of the personal/health data of older PwD; the latter, the need to have clear guidelines when such patients are involved in a study to establish, according to their level of dementia, whether they can independently sign written informed consent letters. As some authors have noted, these are crucial issues regarding vulnerable populations in general, and older PwD require special attention [90] to avoid dehumanized care [91] and to meet the needs for safety/security when using technology for care [81]. Moreover, to assure that the privacy of PwD is respected when using digital devices and to make them feel comfortable with their use, it seems necessary to involve older users with dementia in the decision to adopt monitoring tools, for instance. Also, to assure legal, responsible, and dignified surveillance, devices should be unobtrusive [24,78]. However, as reported in some studies [65], even though ICs do not often perceive an ethical dilemma where the primary need for the safety of PwD is concerned, ethical questions on the impact of technology on their needs, including the possible alteration of such needs to match the available potentiality of current tools, have arisen from several studies without a definitive answer.

### 4.5. Limitations of the Study

This study has some limitations that need to be considered. First, the small number of PwD and ICs cannot be considered as representative of a larger population, and the criteria for sampling saturation were not applied in this respect; thus, this limits the generalizability of the findings. However, it should be pointed out that some studies have allowed small groups of PwD to participate in the co-design process. In particular, some authors [31] have suggested that smaller groups may be organized for co-designing with PwD and have also noted some limitations when involving PwD in design research, e.g., the potential burden on ICs, who might refuse to participate in the study, which, in turn, could lead to refusal on the part of PwD. Moreover, the needs, physical capabilities, and diseases of older people can limit their involvement in co-design [29]; and, in fact, the process cannot include PwD who are not able to express themselves verbally [31]. Other authors [38] have reported involving only one man and one woman living with dementia (in addition to 10 ICs and three social care professionals) in the co-design phase, but this is an appreciable result because few studies have “formally evaluated the experiences of the public and patient participants in the co-design of dementia care interventions” [38]. However, the Alzheimer’s Society [92] recommends involving three to six PwD in dementia-friendly focus groups. One study suggests that “research projects could lead to qualitative interviews with a smaller number of participants while simultaneously asking a larger group to test the web-based decision aid” [93]. 

We would also like to add that in our experience, the focus groups were held when there were still cases of COVID-19; thus, some people were unable to participate because they were ill, even though they had expressed their availability. Concerning the limitations of this study, it should also be considered that the qualitative findings are drawn from a limited number of questions that do not reflect all the possible aspects related to people’s relationship with technological tools. Finally, few quotations were collected from the narratives of PwD because the duration of their sessions was short to avoid potential stress [31] and because they interacted less and there were only two of them. As explained above (Section 3, Section 3.1), this was a consequence of the limited sample size. In addition, it should be pointed out that this study analyzed only the Italian data, to first focus on this country, even though a comparison of the results from focus groups in all the countries involved in the project could have added further insights to the interpretation of the Italian context. The publication of comparative cross-country data will follow.

## 5. Conclusions

The results from the co-design phase of the DemiCare project carried out in Italy, although based on a very small sample, indicate that technology, in general, and the proposed DemiCare integrated system, in particular, could be helpful for ICs, especially for monitoring PwD during the night. However, depending on their level of dementia, PwD sometimes do not accept devices, and ICs may lack the skills required to use digital devices; thus, they would need training in this respect. With regard to the “promising” DemiCare solution, aspects, such as the creation of a network/integration with other available care supports/HPs, personalization relative to responding to different needs/care contexts, comfortable wearability of devices, and ethical/privacy issues linked to their use, seem important for potentially allowing aging in place, i.e., to hopefully support PwD to stay in the community for as long as possible, thus also supporting ICs.

However, these considerations still only represent a hypothesis to be confirmed by further progress in the DemiCare study. Despite this, preliminary insights from co-designing with EUs, especially PwD and HPs, indicate that this approach is a crucial step in developing care interventions. Co-creation, thus, seems to be potentially effective, even though it is a demanding and difficult task to accomplish.

In the future, research should adopt a co-creation approach in the preliminary and periodic evaluation stages of the study design, and it should focus on integrated digital devices to support PwD and their ICs to promote an appropriate user-centered support model.

## Figures and Tables

**Table 1:** Typology of participants and questions.

Participants	Questions
Focus group 1—ICs	What does a typical week look like in terms of daily and care activities?
	When do you experience burden and why?
	Do you need more information on care management?
	From whom (e.g., family and services) do you have support?
	Could technology be useful in supporting caregiving?
Focus group 2—ICs and older PwD	Which smart devices do you use in your daily routine?
	What could convince you to use or not use them?
	What are your opinions/expectations regarding technology?
	What are your opinions/expectations regarding the DemiCare system?
	How can the DemiCare system remind you to use it?
Focus group 3—HPs	What is your first impression of the overall vision of DemiCare for providing smart devices for PwD and app-based guidance for ICs?
	What about the wearability and personalization factors of the devices and app?
	How do you think ICs and PwD could benefit from the DemiCare system? What would motivate you to act in this respect?
	Are you aware of any similar support available on the market?

IC, informal caregiver; PwD, people with dementia; HP, healthcare professional.

**Table 2:** Roles and areas of expertise of HPs.

N.	HPs Involved
1	Psychologist: cognitive disorders, diagnostics, and rehabilitation of older patients
2	Psychologist: cognitive disorders, diagnostics, and training course for family caregivers
3	Psychologist: coordination of home services for PwD and AD care
4	Neuropsychologist: cognitive disorders, diagnostics, and rehabilitation of older patients
5	Biomedical engineer: experience with AAL projects on dementia
6	Health social worker: responsible for social policies in the Marche region (Italy) and support for family caregivers
7	Medical doctor: specialization in psychotherapy

PwD, people with dementia; AD, Alzheimer’s disease; AAL, active and assisted living.

## Data Availability

All the relevant data supporting the findings (numbers of statements and excerpts of transcripts) are within the manuscript. The full qualitative dataset (verbatim transcripts of narratives in Italian) is not publicly available owing to ethical restrictions and privacy issues regarding a vulnerable population (PwD). The data contain potentially identifying or sensitive information that could compromise the anonymity of the research participants (e.g., names of persons and other potentially indirect identifiers of the respondents).

## Supplementary Materials

The following supporting information can be downloaded at https://www.mdpi.com/article/10.3390/healthcare11192640/s1: Table S1: Reporting checklist: Standards for Reporting Qualitative Research (SRQR); Table S2: Reporting checklist: STrengthening the Reporting of OBservational studies in Epidemiology (STROBE); Table S3: Reporting checklist: Guidance for Reporting the Involvement of Patients and the Public (GRIPP2 checklist, short form).

## Abbreviations

AAL	Active and assisted living
AD	Alzheimer’s disease
AI	Artificial intelligence
App	Application
CAQDAS	Computer-assisted qualitative data analysis software
EU	End user
GDPR	General data protection regulation
GPS	Global Positioning System
GRIPP	Guidance for Reporting the Involvement of Patients and the Public
HB	High burden
HP	Healthcare professional
IC	Informal caregiver
ISTAT	Italian National Institute of Statistics
LB	Low burden
LTC	Long-term care
MCI	Mild cognitive impairment
MCW	Migrant care worker
MD	Mild dementia
MoD	Moderate dementia
PwD	People with dementia
SRQR	Standards for Reporting Qualitative Research
STROBE	STrengthening the Reporting of OBservational studies in Epidemiology
WHO	World Health Organization

## References

[1] United Nations (2022). World Population Prospects 2019.

[2] ISTAT (2023). Popolazione Italiana Residente al 1 Gennaio 2023.

[3] EUROSTAT (2022). Population Structure and Ageing.

[4] Katz S. (1983). Assessing Self-Maintenance: Activities of Daily Living, Mobility, and Instrumental Activities of Daily Living. J. Am. Geriatr. Soc..

[5] WHO (2021). Global Status Report on the Public Health Response to Dementia.

[6] Hugo J., Ganguli M. (2014). Dementia and cognitive impairment: Epidemiology, diagnosis, and treatment. Clin. Geriatr. Med..

[7] GBD 2019 Dementia Forecasting Collaborators (2022). Estimation of the global prevalence of dementia in 2019 and forecasted prevalence in 2050: An analysis for the Global Burden of Disease Study 2019. Lancet Public Health.

[8] Alzheimer Europe (2019). Dementia in Europe Yearbook 2019: Estimating the Prevalence of Dementia in Europe.

[9] Harrison K.L., Ritchie C.S., Patel K., Hunt L.J., Covinsky K.E., Yaffe K., Smith A.K. (2019). Care Settings and Clinical Characteristics of Older Adults with Moderately Severe Dementia. J. Am. Geriatr. Soc..

[10] Costa G., Melchiorre M.G., Arlotti M. (2020). Ageing in Place in Different Care Regimes. The Role of Care Arrangements and the Implications for the Quality of Life and Social Isolation of Frail Older People. DAStU Work. Pap. Ser..

[11] Ranci C., Arlotti M., Bernardi L., Melchiorre M.G. (2020). La Solitudine Dei Numeri Ultimi. Abit. Anziani..

[12] Costa G., Ranci C., Pavolini E. (2013). Italy: A case of missing reforms but incremental institutional change in Long Term Care. Reforms in Long Term Care Policies in Europe Investigating Institutional Change and Social Impacts.

[13] Ienca M., Schneble C., Kressig R.W., Wangmo T. (2021). Digital health interventions for healthy ageing: A qualitative user evaluation and ethical assessment. BMC Geriatr..

[14] Ratnayake M., Lukas S., Brathwaite S., Neave J., Henry H. (2022). Aging in Place: Are We Prepared?. Dela. J. Public Health.

[15] Rogers W.A., Ramadhani W.A., Harris M.T. (2020). Defining Aging in Place: The Intersectionality of Space, Person, and Time. Innov. Aging.

[16] Fabricatore C., Radovic D., Lopez X., Grasso-Cladera A., Salas C.E. (2019). When technology cares for people with dementia: A critical review using neuropsychological rehabilitation as a conceptual framework. Neuropsychol. Rehabil..

[17] Kim K.I., Gollamudi S.S., Steinhubl S. (2017). Digital technology to enable aging in place. Exp. Gerontol..

[18] Melchiorre M.G., Papa R., Rijken M., van Ginneken E., Hujala A., Barbabella F. (2018). eHealth in Integrated Care Programs for People with Multimorbidity in Europe: Insights from the ICARE4EU Project. Health Policy.

[19] Smith G.C., Tobin S.S., Robertson-Tchabo E.A., Power P.W. (1995). Strengthening Aging Families: Diversity in Practice and Policy.

[20] Hooker K., Bowman S.R., Coehlo D.P., Lim S.R., Kaye J., Guariglia R., Li F. (2002). Behavioral change in persons with dementia: Relationships with mental and physical health of caregivers. J. Gerontol. B Psychol. Sci. Soc. Sci..

[21] Vitaliano P.P., Zhang J., Scanlan J.M. (2003). Is caregiving hazardous to one’s physical health? A meta-analysis. Psychol. Bull..

[22] Thompson C.A., Spilsbury K., Hall J., Birks Y., Barnes C., Adamson J. (2007). Systematic review of information and support interventions for caregivers of people with dementia. BMC Geriatr..

[23] Okabayashi H., Sugisawa H., Takanashi K., Nakatani Y., Sugihara Y., Hougham G.-W. (2008). A longitudinal study of coping and burnout among Japanese family caregivers of frail elders. Aging Ment. Health.

[24] Vermeer Y., Higgs P., Charlesworth G. (2019). What do we require from surveillance technology? A review of the needs of people with dementia and informal caregivers. J. Rehabil. Assist. Technol. Eng..

[25] Abdi S., de Witte L., Hawley M. (2021). Exploring the Potential of Emerging Technologies to Meet the Care and Support Needs of Older People: A Delphi Survey. Geriatrics.

[26] Guisado-Fernández E., Giunti G., Mackey L.M., Blake C., Caulfield B.M. (2019). Factors influencing the adoption of smart health technologies for people with dementia and their informal caregivers: Scoping review and design framework. JMIR Aging.

[27] Stara V., Rampioni M., Mosoi A.A., Kristaly D.M., Moraru S.A., Paciaroni L., Paolini S., Raccichini A., Felici E., Rossi L. (2022). A Technology-Based Intervention to Support Older Adults in Living Independently: Protocol for a Cross-National Feasibility Pilot. Int. J. Environ. Res. Public Health.

[28] Osborne S.P., Radnor Z., Strokosch K. (2016). Co-production and the co-creation of value in public services: A suitable case for treatment?. Public Manag. Rev..

[29] Sumner J., Chong L.S., Bundele A., Wei Lim Y. (2021). Co-Designing Technology for Aging in Place: A Systematic Review. Gerontologist.

[30] Papa R., Efthymiou A., Lamura G., Piccinini F., Onorati G., Papastavrou E., Tsitsi T., Casu G., Boccaletti L., Manattini A. (2020). Review and Selection of Online Resources for Carers of Frail Adults or Older People in Five European Countries: Mixed-Methods Study. JMIR mHealth uHealth.

[31] Wang G., Marradi C., Albayrak A., van der Cammen T.J. (2019). Co-designing with people with dementia: A scoping review of involving people with dementia in design research. Maturitas.

[32] Hendriks N., Truyen F., Duval E. (2013). Designing with dementia: Guidelines for participatory design together with persons with dementia. Hum. Comput. Interact..

[33] Knopman D.S., Petersen R.C. (2014). Mild cognitive impairment and mild dementia: A clinical perspective. Mayo Clin. Proc..

[34] Davies N., Manthorpe J., Sampson E.L., Lamahewa K., Wilcock J., Mathew R., Iliffe S. (2018). Guiding practitioners through end of life care for people with dementia: The use of heuristics. PLoS ONE.

[35] Folstein M.F., Folstein S.E., McHugh P.R. (1975). Mini-mental state: A practical method for grading the cognitive state of patients for the clinician. J. Psychiatr. Res..

[36] O’Leary K., Tanghe D., Pratt W., Ralston J. (2018). Collaborative Health Reminders and Notifications: Insights from Prototypes. AMIA Annu. Symp. Proc..

[37] Rodgers P.A. (2018). Co-designing with people living with dementia. CoDesign.

[38] Lord K., Kelleher D., Ogden M., Mason C., Rapaport P., Burton A., Leverton M., Downs M., Souris H., Jackson J. (2022). Co-designing complex interventions with people living with dementia and their supporters. Dementia.

[39] Kitzinger J. (1995). Qualitative research. Introducing focus groups. BMJ.

[40] Yeates L., Gardner K., Do J., van den Heuvel L., Fleming G., Semsarian C., McEwen A., Adlard L., Ingles J. (2022). Using co-design focus groups to develop an online COmmunity suPporting familiEs after Sudden Cardiac Death (COPE-SCD) in the young. BMJ Open.

[41] Bamford C., Bruce E., Wilkinson H. (2002). Successes and challenges in using focus groups with older people with dementia. The Perspectives of People with Dementia: Research Methods and Motivations.

[42] Kennedy M.R., Meulen R. (2018). Recommendations for Involving People with Dementia or Mild Cognitive Impairment and Their Informal Caregivers and Relatives in the Assisted Living Project.

[43] Ritchie J., Lewis J. (2003). Qualitative Research Practice. A Guide for Social Science Students and Researchers.

[44] Suárez-Figueroa M.C., Ruckhaus E., López-Guerrero J., Cano I., Cervera Á. Towards the Assessment of Easy-To-Read Guidelines Using Artificial Intelligence Techniques. Proceedings of the Computers Helping People with Special Needs: 17th International Conference, ICCHP 2020.

[45] Blake M. (2007). Formality and Friendship: Research Ethics Review and Participatory Action Research. ACME Int. J. Crit. Geogr..

[46] Goodyear-Smith F., Jackson C., Greenhalgh T. (2015). Co-design and implementation research: Challenges and solutions for ethics committees. BMC Med. Ethics.

[47] European Union (2016). Regulation 2016/679 of the European Parliament and of the Council. General Data Protection Regulation. Off. J. Eur. Union.

[48] Levers M.J.D. (2013). Philosophical Paradigms, Grounded Theory, and Perspectives on Emergence. Sage Open Publ..

[49] Strauss A.L., Corbin J.M. (1998). Basics of Qualitative Research: Techniques and Procedures for Developing Grounded Theory.

[50] Glaser B., Strauss A.L. (1967). The Discovery of Grounded Theory Strategies for Qualitative Research.

[51] Novak J.D. (2001). L’apprendimento Significativo.

[52] Lincoln Y.S., Guba E.G. (1985). Naturalistic Inquiry.

[53] Caspar S., Garschall M., Sfetcu R., Himmelsbach J., Fass F. (2020). SUccessful Caregiver Communication and Everyday Situation Support in dementia care (SUCCESS): Using technology to support caregivers. Alzheimer’s Dement..

[54] VERBI Software (2022). MAXQDA 2022 [Computer Software].

[55] Carcary M. (2011). Evidence Analysis Using CAQDAS: Insights from a Qualitative Researcher. Electron. J. Bus. Res. Methods.

[56] Gibbs G.R. (2014). Using Software in Qualitative Analysis. The Sage Handbook of Qualitative Data Analysis.

[57] O’Brien B.C., Harris I.B., Beckman T.J., Reed D.A., Cook D.A. (2014). Standards for reporting qualitative research: A synthesis of recommendations. Acad. Med..

[58] von Elm E., Altman D.G., Egger M., Pocock S.J., Gøtzsche P.C., Vandenbroucke J.P. (2008). STROBE Initiative. The Strengthening the Reporting of Observational Studies in Epidemiology (STROBE) statement: Guidelines for reporting observational studies. J. Clin. Epidemiol..

[59] Staniszewska S., Brett J., Simera I., Seers K., Mockford C., Goodlad S., Altman D.G., Moher D., Barber R., Denegri S. (2017). GRIPP2 reporting checklists: Tools to improve reporting of patient and public involvement in research. BMJ.

[60] Schulz R., Eden J. (2016). Families Caring for an Aging America.

[61] Grynne A., Browall M., Fristedt S., Ahlberg K., Smith F. (2021). Integrating perspectives of patients, healthcare professionals, system developers and academics in the co-design of a digital information tool. PLoS ONE.

[62] Biderman A., Carmel S., Amar S., Bachner Y.G. (2021). Care for caregivers- a mission for primary care. BMC Fam. Pract..

[63] González-Fraile E., Ballesteros J., Rueda J.R., Santos-Zorrozúa B., Solà I., McCleery J. (2021). Remotely delivered information, training and support for informal caregivers of people with dementia. Cochrane Database Syst. Rev..

[64] Schulz R., Beach S.R., Czaja S.J., Martire L.M., Monin J.K. (2020). Family Caregiving for Older Adults. Annu. Rev. Psychol..

[65] Sriram V., Jenkinson C., Peters M. (2019). Informal carers’ experience of assistive technology use in dementia care at home: A systematic review. BMC Geriatr..

[66] Steiner V., Pierce L.L., Salvador D. (2016). Information Needs of Family Caregivers of People with Dementia. Rehabil. Nurs..

[67] Peterson K., Hahn H., Lee A.J., Madison C.A., Atri A. (2016). In the Information Age, do dementia caregivers get the information they need? Semi-structured interviews to determine informal caregivers’ education needs, barriers, and preferences. BMC Geriatr..

[68] Sixsmith A., Gutman G. (2013). Technologies for Active Aging.

[69] Popescu D., Rusu D., Bacali L., Popescu S. (2018). Multi-Layered Functional Analysis for Smart Homes Design. Procedia Soc. Behav. Sci..

[70] Cobo A., Villalba-Mora E., Pérez-Rodríguez R., Ferre X., Rodríguez-Mañas L. (2021). Unobtrusive Sensors for the Assessment of Older Adult’s Frailty: A Scoping Review. Sensors.

[71] Sharma N., Brinke J.K., Van Gemert-Pijnen J.E.W.C., Braakman-Jansen L.M.A. (2021). Implementation of Unobtrusive Sensing Systems for Older Adult Care: Scoping Review. JMIR Aging.

[72] Pot A.M., Willemse B.M., Horjus S. (2012). A pilot study on the use of tracking technology: Feasibility, acceptability, and benefits for people in early stages of dementia and their informal caregivers. Aging Ment. Health.

[73] Hill J.R., Min E.E., Abebe E., Holden R.J. (2023). Telecaregiving for dementia: A mapping review of technological and non-technological interventions. Gerontologist.

[74] Liu L., Cruz A.M., Ruptash T., Barnard S., Juzwishin D. (2017). Acceptance of global positioning system (GPS) technology among dementia clients and family caregivers. J. Technol. Hum. Serv..

[75] Jelcic N., Agostini M., Meneghello F., Bussè C., Parise S., Galano A., Tonin P., Dam M., Busse C. (2014). Feasibility and efficacy of cognitive telerehabilitation in early Alzheimer’s disease: A pilot study. Clin. Interv. Aging.

[76] Hanson E., Magnusson L., Arvidsson H., Claesson A., Keady J., Nolan M. (2007). Working together with persons with early stage dementia and their family members to design a user-friendly technology-based support service. Dementia.

[77] Berridge C., Turner N.R., Liu L., Karras S.W., Chen A., Fredriksen-Goldsen K., Demiris G. (2022). Advance Planning for Technology Use in Dementia Care: Development, Design, and Feasibility of a Novel Self-administered Decision-Making Tool. JMIR Aging.

[78] McCabe L., Innes A. (2013). Supporting safe walking for people with dementia: User participation in the development of new technology. Gerontechnology.

[79] Landau R., Auslander G.K., Werner S., Shoval N., Heinik J. (2011). Who should make the decision on the use of GPS for people with dementia?. Aging Ment. Health.

[80] Stara V., Vera B., Bolliger D., Paolini S., de Jong M., Felici E., Koenderink S., Rossi L., Von Doellen V., di Rosa M. (2021). Toward the Integration of Technology-Based Interventions in the Care Pathway for People with Dementia: A Cross-National Study. Int. J. Environ. Res. Public Health.

[81] Olsson A., Engström M., Skovdahl K., Lampic C. (2012). My, your and our needs for safety and security: Relatives’ reflections on using information and communication technology in dementia care. Scand. J. Caring Sci..

[82] Rettinger L., Zeuner L., Werner K., Ritschl V., Mosor E., Stamm T., Haslinger-Baumann E., Werner F. A mixed-methods evaluation of a supporting app for informal caregivers of people with dementia. Proceedings of the 13th ACM International Conference on PErvasive Technologies Related to Assistive Environments.

[83] Bajenaru L., Ianculescu M., Dobre C. (2018). A Holistic Approach for Creating a Digital Ecosystem Enabling Personalized Assistive Care for Elderly, 16th International Conference on Embedded and Ubiquitous Computing.

[84] Wan L., Muller C., Randall D., Wulf V. (2016). Design of a GPS monitoring system for dementia care and its challenges in academia-industry project. ACM Trans. Comput. Interact..

[85] Nishiura Y., Nihei M., Nakamura-Thomas H., Inoue T. (2021). Effectiveness of using assistive technology for time orientation and memory, in older adults with or without dementia. Disabil. Rehabil. Assist. Technol..

[86] Behera C.K., Condell J., Dora S., Gibson D.S., Leavey G. (2021). State-of-the-art sensors for remote care of people with dementia during a pandemic: A systematic review. Sensors.

[87] AlQudah A.A., Al-Emran M., Shaalan K. (2021). Technology Acceptance in Healthcare: A Systematic Review. Appl. Sci..

[88] Guo Y., Liu X., Peng S., Jiang X., Xu K., Chen C., Wang Z., Dai C., Chen W. (2020). A review of wearable and unobtrusive sensing technologies for chronic disease management. Comput. Biol. Med..

[89] Wrede C., Braakman-Jansen A., van Gemert-Pijnen L. (2022). How to create value with unobtrusive monitoring technology in home-based dementia care: A multimethod study among key stakeholders. BMC Geriatr..

[90] Moll S., Wyndham-West M., Mulvale G., Park S., Buettgen A., Phoenix M., Fleisig R., Bruce E. (2020). Are you really doing ‘codesign’? Critical reflections when working with vulnerable populations. BMJ Open.

[91] Hastall M.R., Eiermann N.D., Ritterfeld U. (2014). Formal and informal carers’ views on ICT in dementia care: Insights from two qualitative studies. Gerontechnology.

[92] Alzheimer’s Society (2023). Dementia-Friendly Focus Groups.

[93] Bogza L., Patry-Lebeau C., Farmanova E., Witteman H.O., Elliott J., Stolee P., Hudon C., Giguere A.M.C. (2020). User-Centered Design and Evaluation of a Web-Based Decision Aid for Older Adults Living with Mild Cognitive Impairment and Their Health Care Providers: Mixed Methods Study. J. Med. Internet. Res..

