# Supplementary material for: Personalized Technological Support for Informal Caregivers of Older People with Dementia: A Co-Design Approach Involving Potential End Users and Healthcare Professionals in Three Focus Groups in Italy
Source: Healthcare (Basel). 2023 Sep 28;11(19):2640. doi: 10.3390/healthcare11192640 (PMC10572801; doi:10.3390/healthcare11192640)
Supplement: Supplementary file 1 [file healthcare-11-02640-s001.zip › Table S2. Checklist STROBE.pdf]

Table S2: STrengthening the Reporting of OBservational studies in Epidemiology (STROBE)

Paper: *"Personalized technological supports for informal caregivers of older people with dementia: a co-design approach involving potential end-users and healthcare professionals in three focus groups in Italy".*

	Item No.	Recommendation	Page No.	Relevant text from manuscript
**Title and abstract**	1	(*a*) Indicate the study's design with a commonly used term in the title or the abstract	1	Co-design and focus groups (in the Title and Abstract)
		(*b*) Provide in the abstract an informative and balanced summary of what was done and what was found	1	The study aimed to analyse the opinions of end-users (EUs), i.e., older people with dementia (PwD) aged 65 years and over, their informal caregivers (ICs), and healthcare professionals (HPs), with respect to the use of digital technologies to support care activities. Qualitative data were collected during the co-design phase of the European project DemiCare. The paper focused on the Italian context. Three focus groups were carried out in April-June 2022. Qualitative data were analysed by using MaxQDA software. Smart devices seem to be positively considered by ICs and HPs, although difficulty of technology acceptance by older PwD emerged.
**Introduction**				
Background/rationale	2	Explain the scientific background and rationale for the investigation being reported	1-3	Ageing is particularly demanding when people become frail, vulnerable and disabled, with several difficulties in performing the activities of daily living, especially for older people with dementia. Dementia affects both the older cared for and their informal/family caregivers, since usually 64% of the

	Item No.	Recommendation	Page No.	Relevant text from manuscript
				former group receive assistance at home. Older PwD and their families can have a great support from digital solutions. In this respect it seems necessary to analyse the perspective of EUs, in order to develop technologies tailored to their needs. For this reason, innovative research projects include a co-design phase aiming at understanding their exigencies.
Objectives	3	State specific objectives, including any prespecified hypotheses	3	1) Which are the main caregiving activities of ICs of PwD and which support is available? 2) Which is the current context of use of digital devices by ICs and PwD, and the potential of available technology? 3) Which are the opinions/expectations of ICs, PwD and HPs, regarding the DemiCare system? 4) Which personalization factors of devices could overall improve their acceptance and wearability?
**Methods**				
Study design	4	Present key elements of study design early in the paper	3-4	Results reported in this paper come from the Italian co-design phase of the European research project 'DemiCare'. The whole study started in 2022 and is still running. The overall project involves four countries, i.e., Austria, Italy, Romania, and The Netherlands. It is targeting ICs (aged 18 years and over), and respective cared for with MCI or MD (the latter as Mini-Mental State Examination – MMSE - ranging 20-25), living at home and aged 65 years and over. The main DemiCare study aims at testing a personalized digital solution, by means of

	Item No.	Recommendation	Page No.	Relevant text from manuscript
				smart soles and smartwatches used by the care recipients, and a personalized App for smartphone used by the IC. In order to achieve effective personalized supports and tools, a co-design/co-creation phase has been carried out by means of focus groups with ICs, older PwD, and HPs or other experts in the care sector (e.g., legal/ethics experts). Focus groups in qualitative research represent an appropriate method to involve participants in a co-design approach, especially with regard to PwD.
Setting	5	Describe the setting, locations, and relevant dates, including periods of recruitment, exposure, follow-up, and data collection	4-5	The subjects involved in the three focus groups were overall recruited in spring-summer 2022. Older PwD and respective ICs were included in two focus groups dedicated to EUs. In Italy (Ancona city, Marche region), the country whose results are reported in this paper, these two focus groups were carried out in April-May 2022: the first online and only with two ICs; the second with two ICs and respective PwD on site. EUs have been recruited with the help of four psychologists from the 'Neurology/Alzheimer's Centre/Stroke Unit, and Research Centre for Neurological Diseases of Older People' of the National Institute of Health and Science on Aging (IRCCS INRCA). HPs in the care sector, involved in the third focus group, were recruited by the respective research team members, drawing from their own extensive network of relevant expertise. In Italy seven HPs (three

	Item No.	Recommendation	Page No.	Relevant text from manuscript
				psychologists, one neuropsychologist, one biomedical engineer, one health-social worker, and one medical doctor) were considered sufficiently representative and involved in an online focus group in June 2022.
Participants	6	(*a*) *Cohort study*—Give the eligibility criteria, and the sources and methods of selection of participants. Describe methods of follow-up<br>*Case-control study*—Give the eligibility criteria, and the sources and methods of case ascertainment and control selection. Give the rationale for the choice of cases and controls<br>***Cross-sectional study*—Give the eligibility criteria, and the sources and methods of selection of participants**	4	A non-probability sampling technique (purposive sample) was used, with individuals selected for their characteristics (inclusion criteria mentioned above: ICs aged 18 years and over, respective cared for with MCI or MD, living at home and aged 65 years and over.) which allow a good exploration of the themes of the study.
		(*b*) *Cohort study*—For matched studies, give matching criteria and number of exposed and unexposed<br>*Case-control study*—For matched studies, give matching criteria and the number of controls per case		
Variables	7	Clearly define all outcomes, exposures, predictors, potential confounders, and effect modifiers. Give diagnostic criteria, if applicable	5-6<br>Table 2	The two co-design focus groups with ICs and older PwD cared for aimed at exploring the context of use of potential digital solutions (e.g., devices for supporting care activities and need for information in this regard). Also, the crucial aspect of unobtrusive but effective reminder mechanisms, ensuring that PwD accept and do not forget to wear and charge the smart devices, has been considered. In particular, smart devices already in use (e.g., smartphone, tablet), and a first impression on DemiCare App and overall solution/vision, were investigated (e.g., as potential impact on the relationship between carer and PwD). In the third

	Item No.	Recommendation	Page No.	Relevant text from manuscript
				co-design focus group, preliminary insights from previous ones were discussed and deepened by HPs, focusing on wearability and personalization approaches/factors (e.g., IC background about the level of knowledge of dementia; environment as available local care providers/supports), which could influence the information needed from different ICs. Moreover, challenges regarding the willingness of PwD to be monitored and to use smart devices (such as smartwatches or smart soles) were explored.
Data sources/ measurement	8*	For each variable of interest, give sources of data and details of methods of assessment (measurement). Describe comparability of assessment methods if there is more than one group	7	The qualitative data were analysed by applying an open coding process. This is an aspect allowing in turn the authors to analyse the contents of the focus groups by adopting the constant comparison technique. The transcribed narratives were read by three researchers independently, and the contents were codified in order to highlight concepts raised from the focus groups. The subthemes emerged from the analytical process were then grouped into the codes referring to the same phenomenon, according to their similarities. These were subsequently grouped into overarching/higher-order themes, that finally were described in a conceptual map, in order to provide a clear description of the findings. Some relevant quotations are included, with codes indicating only the role in the focus group (PwD, IC, and HP) and progressive numeration of participants, to ensure

	Item No.	Recommendation	Page No.	Relevant text from manuscript
				the de-identification of excerpts. The analysis was conducted with the support of MAXQDA 2020 software (VERBI Software, Berlin, Germany), one of the available Computer Assisted Qualitative Data Analysis Software (CAQDAS) packages, in order to make the analytical process more flexible and effective. We followed the Standards for Reporting Qualitative Research [SRQRreporting guidelines] (S1 File), according to O'Brien and colleagues.
Bias	9	Describe any efforts to address potential sources of bias	17-18<br>7	Our study was exploratory only, with a small sample that cannot be considered representative of the target population. However, the trustworthiness of the qualitative analysis/results follows fundamental criteria according to Lincoln and Guba, i.e., credibility (use of a topic guide partly based on questionnaires applied in previous studies on PwD), analytic transferability (preliminary literature review as background data), dependability and confirmability (detailed description of the study protocol, use of replicable methods, and collaborative discussion with colleagues).
Study size	10	Explain how the study size was arrived at	5	Unfortunately, few PwD (2) and ICs (3) were overall recruited, following several difficulties encountered in involving them in the study, as reported also by previous literature, and thus criteria for sampling saturation in this respect were not applied, as explained better in the Limitations section (p. 17)

	Item No.	Recommendation	Page No.	Relevant text from manuscript
Quantitative variables	11	Explain how quantitative variables were handled in the analyses. If applicable, describe which groupings were chosen and why	N.A.	
Statistical methods	12	(*a*) Describe all statistical methods, including those used to control for confounding	N.A.	
		(*b*) Describe any methods used to examine subgroups and interactions	N.A.	
		(*c*) Explain how missing data were addressed	N.A.	
		(*d*) *Cohort study*—If applicable, explain how loss to follow-up was addressed<br>*Case-control study*—If applicable, explain how matching of cases and controls was addressed<br>***Cross-sectional study*—If applicable, describe analytical methods taking account of sampling strategy**	N.A.	
		(*e*) Describe any sensitivity analyses	N.A.	
**Results**				
Participants	13*	(a) Report numbers of individuals at each stage of study—eg numbers potentially eligible, examined for eligibility, confirmed eligible, included in the study, completing follow-up, and analysed	4-6; 8	Two ICs in the first focus group. Two ICs and two PwD in the second focus group. Seven HP in the third focus group. One IC participated in both focus groups, as allowed by the overall study design, to maintain (when possible) the continuity of collaboration with EUs throughout the whole co-design phase (and project). Among HPs: three psychologists, one neuropsychologist, one biomedical engineer, one health-social worker, one medical doctor.
		(b) Give reasons for non-participation at each stage	17	Focus groups were carried out when there were still cases of COVID-19 pandemic, and thus some participants were unable to participate because they were ill, even though they had expressed their availability in this respect.

	Item No.	Recommendation	Page No.	Relevant text from manuscript
		(c) Consider use of a flow diagram	N.A.	Our study was exploratory only, with a small sample.
Descriptive data	14*	(a) Give characteristics of study participants (e.g. demographic, clinical, social) and information on exposures and potential confounders	8<br>Table 1<br>Table 3	ICs (two sons and one wife), are aged 51-69 years, married and living with the respective spouse. They are both workers and housewives. Two PwD are aged 73 and 85 years, both retired, one living with the spouse and one living alone, one affected by MD and one by moderate dementia (MoD). Even though eligibility criteria of the study aimed at including subjects with MD, for the purpose of the co-design phase also a moderate level was considered useful and thus recruited. Overall, both genders participated in research on an equal footing (Table 3).
		(b) Indicate number of participants with missing data for each variable of interest	N.A.	
		(c) *Cohort study*—Summarise follow-up time (eg, average and total amount)		
Outcome data	15*	*Cohort study*—Report numbers of outcome events or summary measures over time		
		Case-control study—Report numbers in each exposure category, or summary measures of exposure		
		***Cross-sectional study*—Report numbers of outcome events or summary measures**	8-9<br>Fig-1	The qualitative data collected from ICs, older PwD and HPs were analysed by the identification of the main themes and subthemes. Then, these were summarized in a conceptual map (Figure 1 cited at p. 16). On the whole, the analysis made it possible to classify the results emerging from the three focus groups into 187 main citations (also several statements by each respondent) relating to seven

	Item No.	Recommendation	Page No.	Relevant text from manuscript
				main thematic areas (and further subthemes): daily activities (11), care tasks (21), information needs (7), supports received (13), relationships with and expectations from technology (32), functionality of the DemiCare integrated system (94), and ethics issues referred by both ICs (2 citations) and HPs (7 citations). Also, care activities were classified according to the related high (HB) or low level (LB) of burden for the ICs, as suggested by some authors.
Main results	16	(*a*) Give unadjusted estimates and, if applicable, confounder-adjusted estimates and their precision (eg, 95% confidence interval). Make clear which confounders were adjusted for and why they were included	9-14	ICs do not have enough time for general daily activities and for themselves, due to demanding care tasks, with consequent difficulty in reconciling own life and caring for PwD. ICs also refer some information needs, on available care supports services, on possible technological supports which could be of help in this respect.<br>The symptoms of PwD seem to impact and hinder/obstacle their acceptance and use of technological tools, especially when they are not easy to use. The older person with MoD, does not perceive the technology as useful, and is not interested in testing the DemiCare system. The older person with MD is very interested in the DemiCare solution and very curious to try it.<br>The HPs indicate that the DemiCare system could benefit from integration/collaboration with other available support services. The most appreciate functionality of DemiCare is the personalization of the service proposed, that is an ad hoc/appropriate

	Item No.	Recommendation	Page No.	Relevant text from manuscript
				support for the specific needs of both PwD and ICs. Opinions on the wearability of smart devices for generally focused on easy to use, comfort, dimension, charging frequency of the batter.
		(*b*) Report category boundaries when continuous variables were categorized	Table 3	MMSE level of older people: Mild/Early (MMSE 20-25); Moderate (MMSE 10-20)
		(*c*) If relevant, consider translating estimates of relative risk into absolute risk for a meaningful time period	N.A.	
Other analyses	17	Report other analyses done—eg analyses of subgroups and interactions, and sensitivity analyses	N.A.	
**Discussion**				
Key results	18	Summarise key results with reference to study objectives	14-17	The aim of this study was to involve ICs, older PwD cared for, and HPs, in the co-design phase of the DemiCare research project, in order to explore the potential interest in the use of smart devices for supporting caregiving activities. Moreover, the opinions with respect to wearability and personalization of the devices which could be used in daily living (e.g., smartwatch and smart sole for PwD cared for, and an App for smartphone for IC) were explored. Results showed overall that smart devices seem to be positively accepted by ICs and HPs, although limitations regarding difficulty of acceptance of technology by PwD emerged. The opinion of EUs on technology, and their individual characteristics, are thus particularly important. Facilitating collaboration among researchers, HPs and patients, in co-designing relevant information

	Item No.	Recommendation	Page No.	Relevant text from manuscript
				meeting the EUs' needs, can have a potential constructive impact on the development of digital devices.
Limitations	19	Discuss limitations of the study, taking into account sources of potential bias or imprecision. Discuss both direction and magnitude of any potential bias	17	The small number of PwD and ICs participants cannot be considered as representative of a larger population, and criteria for sampling saturation in this respect were not applied, thus these aspects limits the generalization of the findings. However, it is to highlight that some literature allows small groups of PwD in co-design. In particular, Wang and colleagues indicate some limitations of involving PwD in design research, e.g., the potential burden of the IC who could thus refuse to participate in the study, and this in turn could lead to a refuse also of the PwD. Moreover, older adults' needs, their physical capabilities and diseases, can limit their involvement in co-design, and in particular co-design cannot include PwD who are not able to express themselves verbally. Other authors also report to have involved in a co-design phase only one man and one woman living with dementia (in addition to 10 ICs and three social care professionals), but this is however an appreciable result since few studies have 'formally evaluated the experiences of public and patient participants in co-design of dementia care interventions'. It is also to be considered that the qualitative findings are drawn from a limited number of questions which do not reflect all the possible aspects related to the relationship with

	Item No.	Recommendation	Page No.	Relevant text from manuscript
				technological tools. Finally, few quotations were selected from narratives of PwD, since the duration of sessions with them was short, in order to avoid their potential stress.
Interpretation	20	Give a cautious overall interpretation of results considering objectives, limitations, multiplicity of analyses, results from similar studies, and other relevant evidence	18	In the light of burdening care activities referred by ICs of older PwD, technology in general, and the DemiCare integrated system in particular, could be of help, especially for monitoring the older relatives during the night. However, PwD sometimes do not accept devices, depending on their level of dementia, and also ICs can have low digital skills, thus needing training in this respect. With regard to the 'promising' DemiCare solution, aspects such as to create a network/integration with other available care supports/HPs, personalization factors to respond to different needs/care contexts, in addition to comfortable wearability of devices and ethics issues/privacy linked to their use, seem important for allowing ageing in place, i.e., for supporting PwD to stay as longer as possible in the community, thus supporting in turn ICs. All this information interestingly come from co-designing with ICs, older PwD cared for and HPs, that emerged as a crucial step with a potential positive impact on the process aiming at developing an effective DemiCare integrated solution. Despite the few number of PwD and ICs involved in the study, an exploratory co-creation seems however beneficial for allowing firstly social interactions/connections between participants

	Item No.	Recommendation	Page No.	Relevant text from manuscript
				in the study, and then for facilitating their social inclusion, with a positive impact especially on the subjective well-being of PwD and on the whole design process. In particular, co-designing process with PwD and listening their preferences, thoughts and emotions, even though representing a demanding and difficult task/aim to be realized, is very important and effective.
Generalisability	21	Discuss the generalisability (external validity) of the study results	17<br>7	The small number of PwD and ICs participants cannot be considered as representative of a larger population, and criteria for sampling saturation in this respect were not applied, thus these aspects limits the generalization of the findings. (However) The trustworthiness of the qualitative analysis/results however follows fundamental criteria according to Lincoln and Guba, i.e., credibility (use of a topic guide partly based on questionnaires applied in previous studies on PwD), analytic transferability (preliminary literature review as background data), dependability and confirmability (detailed description of the study protocol, use of replicable methods, and collaborative discussion with colleagues).
**Other information**				
Funding	22	Give the source of funding and the role of the funders for the present study and, if applicable, for the original study on which the present article is based	19	This study is part of the DemiCare project co-financed by the EU Active and Assisted Living Program http://www.aal-europe.eu/ (Grant Agreement Number: AAL-2021-8-169-CP). This work has also partially been supported by the Ricerca Corrente funding from the Italian

Item No.	Recommendation	Page No.	Relevant text from manuscript
			Ministry of Health to IRCCS INRCA. The funders had no role in the design of the study; in the collection, analysis, or interpretation of data; in the writing of the manuscript, or in the decision to publish the results.

*Give information separately for cases and controls in case-control studies and, if applicable, for exposed and unexposed groups in cohort and cross-sectional studies.
Note: An Explanation and Elaboration article discusses each checklist item and gives methodological background and published examples of transparent reporting.
The STROBE checklist is best used in conjunction with this article. Information on the STROBE Initiative is available at www.strobe-statement.org.
