# Supplementary material for: Personalized Technological Support for Informal Caregivers of Older People with Dementia: A Co-Design Approach Involving Potential End Users and Healthcare Professionals in Three Focus Groups in Italy
Source: Healthcare (Basel). 2023 Sep 28;11(19):2640. doi: 10.3390/healthcare11192640 (PMC10572801; doi:10.3390/healthcare11192640)
Supplement: Supplementary file 1 [file healthcare-11-02640-s001.zip › Table S3. Checklist GRIPP2.pdf]

Table S3: Guidance for Reporting Involvement of Patients and the Public (GRIPP2 checklist short form)

Paper: *"Personalized technological supports for informal caregivers of older people with dementia: a co-design approach involving potential end-users and healthcare professionals in three focus groups in Italy"*

Section and topic	Item	Reported on page No
1: Aim	Report the aim of PPI in the study	1-3
2: Methods	Provide a clear description of the methods used for PPI in the study	3-6
3: Study results	Outcomes—Report the results of PPI in the study, including both positive and negative outcomes	9-12
4: Discussion and conclusions	Outcomes—Comment on the extent to which PPI influenced the study overall. Describe positive and negative effects	14, 18
5: Reflections/critical perspective	Comment critically on the study, reflecting on the things that went well and those that did not, so others can learn from this experience	18

PPI=patient and public involvement
